# Supplementary material for: Nutraceuticals silybin B, resveratrol, and epigallocatechin-3 gallate-bind to cardiac muscle troponin to restore the loss of lusitropy caused by cardiomyopathy mutations in vitro, in vivo, and in silico
Source: Front Physiol. 2024 Dec 13;15:1489439. doi: 10.3389/fphys.2024.1489439 (PMC11672104; doi:10.3389/fphys.2024.1489439)
Supplement: Supplementary file 1 [file DataSheet1.pdf]

SUPPLEMENTARY INFORMATION

Nutraceuticals Silybin B, resveratrol and epigallocatechin-3 gallate (EGCG) bind to cardiac muscle troponin to restore the loss of lusitropy caused by cardiomyopathy mutations *in vitro, in vivo*, and *in silico*

Yang, Sheehan, Messer, Tsui , Sparrow, Redwood, Kren, Gould, Marston

CONTENTS:

1 Single [Ca2+] screen for recoupling

2 Many compounds re-couple many mutations.

3 Effects of dobutamine on cardiac myocyte contractility.

4 Lusitropy and the effect of small molecules measured in cardiomyocytes

5 Preferred structure of small molecules with atoms and rings labelled.

6 Comparison of the effects of EGCG and ECG on the distribution of helix A/B and interdomain angles.

7 CCPtraj analysis of ligand binding, ligand hotspots on representative structures, link to movies

8 snapshots from single 1500ns MD trajectories, 7500 total frames

SUPPLEMENT 1

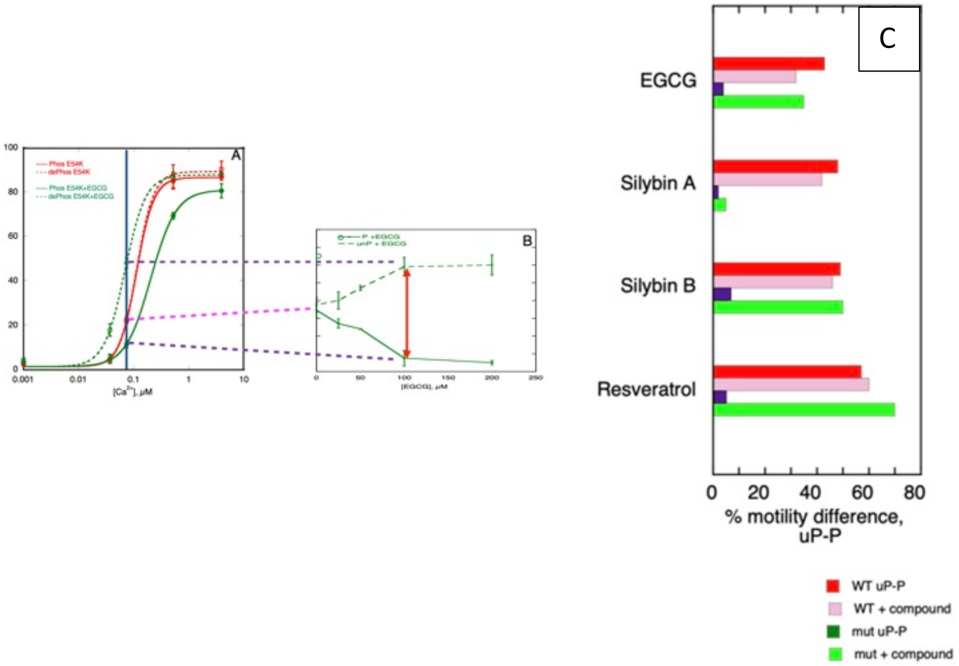

A, B Single Ca2+ concentration screen for Ca2+ sensitivity shifts and recoupling

Screening principle. (A) The effects of EGCG on Ca2+ regulation of E54K-containing thin filaments. (B) EGCG dose response data of thin filament motility measured at a constant Ca2+ concentration corresponding to the Ca2+ EC50 for mutant filament mobility. Red lines with points represent mutation with no drug and Green lines with points represent mutation treatment with EGCG (100μM). Solid lines with points represent phosphorylated TnI and broken lines with points represent unphosphorylated TnI. Purple and pink broken lines illustrate the relationship between thin filament motility at a constant intermediate Ca2+ concentration.

C Fixed [Ca^{2+}] assay (~EC_{50}) using WT or TPM1 E180G HCM mutant .

The increased motility due to dephosphorylation is plotted as described above. Small molecules do not affect wild-type response (red and pink bars). Thin filaments containing E180G tropomyosin show no increase in motility on phosphorylation (Black bars) but EGCG, silybin B and resveratrol restore the dephosphorylation effect to E180G whilst silybin A does not (green bars).

SUPPLEMENT 2
Ability of small molecules to recouple thin filaments with many different uncoupling mutations.

	EGCG	ECG	SA	SB	resveratrol
TPM1 R180G	+	-	-	+	+
TNNC1 G159D	+		-	+	
ACTC E99K	+		-	+	+
TPM1 E54K	+		-	+	
MYBPC3 R820W	+				
TNNT2 R92Q	+		-	+	+
TNNT2 K280N	+				
TNNT2 Δ28	+				
TNNT2 K2732N	+				
TNNT2 S179F	+				
TNNT2 ΔE160	+				
ACTC E361G	+*			+*	

\+ mutant is recoupled, - mutant is not recoupled
Black, tested by Single point IVMA assay
Red, tested by IVMA and in myocytes, * tested in papillary muscle

Data reproduced from Sheehan et al, (2018); Messer et al (2016); Sheehan PhD thesis (Imperial College London, 2019) and the work in this paper.

SUPPLEMENT 3

A

Effects of dobutamine on cardiac myocyte contractility.

	n	Amplitude Raw Data, *μm*	sem	T_{90} contraction, sec	sem	T_{90} relaxation, sec	sem
**mouse WT**	57	3.566	*2.427*	0.046	*0.014*	0.168	*0.052*
**mouse WT+ Dob**	38	5.27	*3.08*	0.044	*0.010*	0.134	*0.037*
**Mouse E99K**	58	3.593	*2.06*	0.065	*0.018*	0.246	*0.08*
**Mouse E99K+ Dob**	43	5.195	*3.0*	0.066	*0.02*	0.277	*0.086*
**GP WT**	64	4.25	*1.24*	0.102	*0.009*	0.464	*0.057*
**GP WT + Dob**	45	4.12	*1.43*	0.0883	*0.006*	0.365	*0.042*
**GP R92Q**	45	3.0	*0.86*	0.98	*0.019*	0.466	*0.077*
**GP R92Q + Dob**	40	3.56	*0.81*	0.102	*0.014*	0.428	*0.061*

B

±dob student	mouse	e99k	GP	R92Q
Amp, %	0.0024	0.0045	0.27	0.35
ttp90	0.628	0.863	0.28	0.88
ttb90	<.0001	0.148	0.028	0.22

Contraction of isolated myocytes was measured. The Incubation medium was Krebs-Hensleit buffer with added 1mM $CaCl_2$, oxygenated with 95% oxygen/5% CO_2. The myocytes were incubated at 37° and continuously stimulated a 1Hz. 10 seconds of contractility was collected and analysed for each cell.

A Contractility in each dish of myocytes was measured in the absence and then presence of 0.4μM dobutamine and 50nM of the specific β2 antagonist, ICI 118,551.

B Changes were analysed by a paired t-test.

SUPPLEMENT 4

Lusitropy and the effect of small molecules measured in cardiomyocytes

		lusitropy	sem	student t		cells/ hearts
**mouse**	WT	-0.20200	0.037000	0.00024000		38/17
	Mut	0.12600	0.029000	0.0015000		30/16
	mut SB	-0.25400	0.044000	0.00020000		8/3
	mut Resv	-0.33100	0.047000	0.0010700		8/3
	mut SA	-0.038000	0.055000	0.54000		6/2
	mut EGCG	-0.22200	0.020000	0.00017000		8/6
**guinea pig**	WT	-0.23700	0.038000	0.00042000		120/8
	Mut	0.091000	0.056000	0.18400		85/5
	mut SB	-0.13000	0.065000	0.0059000		80/5
	mut Resv	-0.17000	0.028000	0.050000		64/4
	mut SA	0.18660	0.080000	0.14800		50/3
	mut EGCG	-0.17000	0.060000	0.054000		51/3

Lusitropy ± sem is given with the number of hearts and total number of cells analysed shown. The significance of lusitropy compared to zero was analysed by a paired t-test.

SUPPLEMENT 5

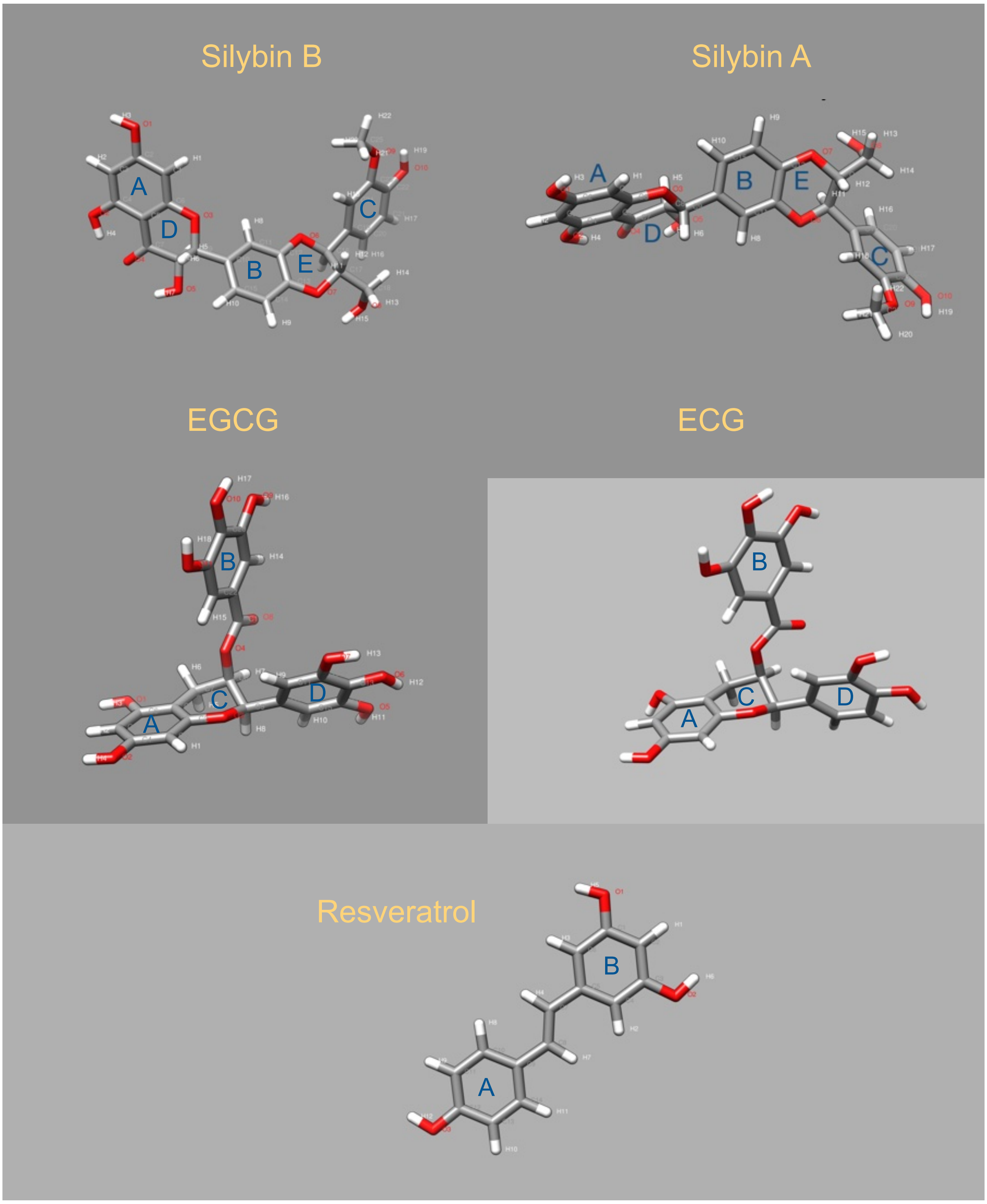

Preferred structure of small molecules after parameterisation is shown.
Atoms and rings are labelled according to the standard protocols.

SUPPLEMENT 6

Comparison of the effects of EGCG and ECG on the distribution of helix A/B and interdomain angles.

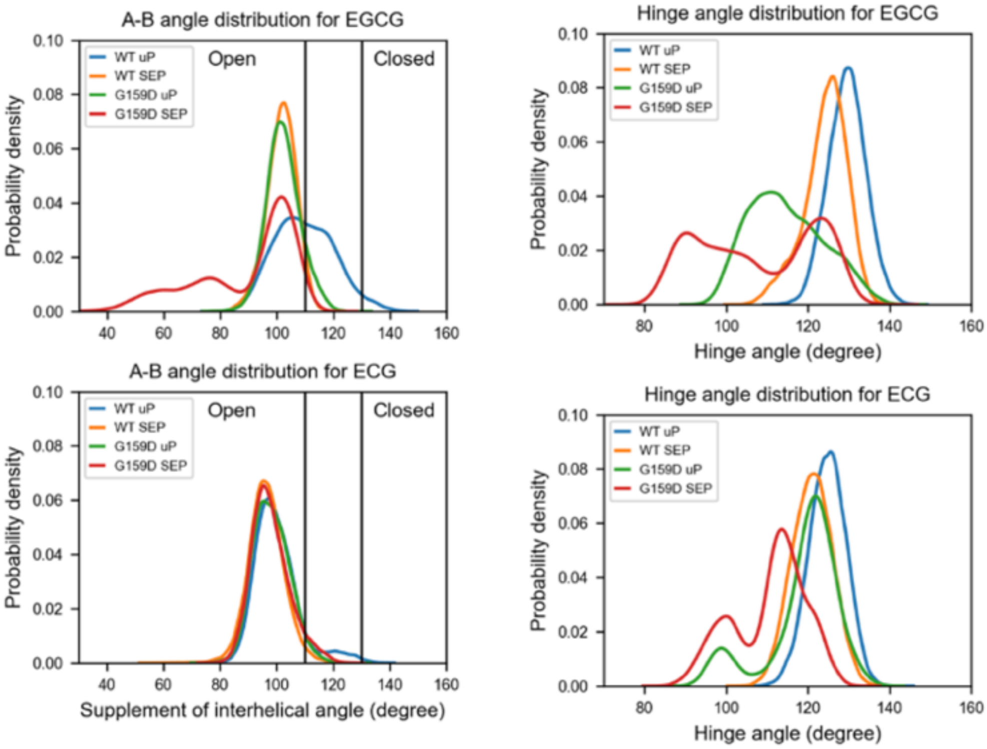

A/B angle: ECG loses the bimodal features of EGCG (both wt and G159D), compatible with its single (desensitising) function
Hinge angle : EGCG loses peaks at 90 in SEP and uP, peaks around 120 dominate and peaks around100-110 diminished ,
especially in uP. In effect a reduction in complexity, compatible with loss of the recoupling function. It would be interesting to

SUPPLEMENT 7

A CCPtraj analysis of ligand binding,

B   ligand hotspots on representative structures,

C movies

7A

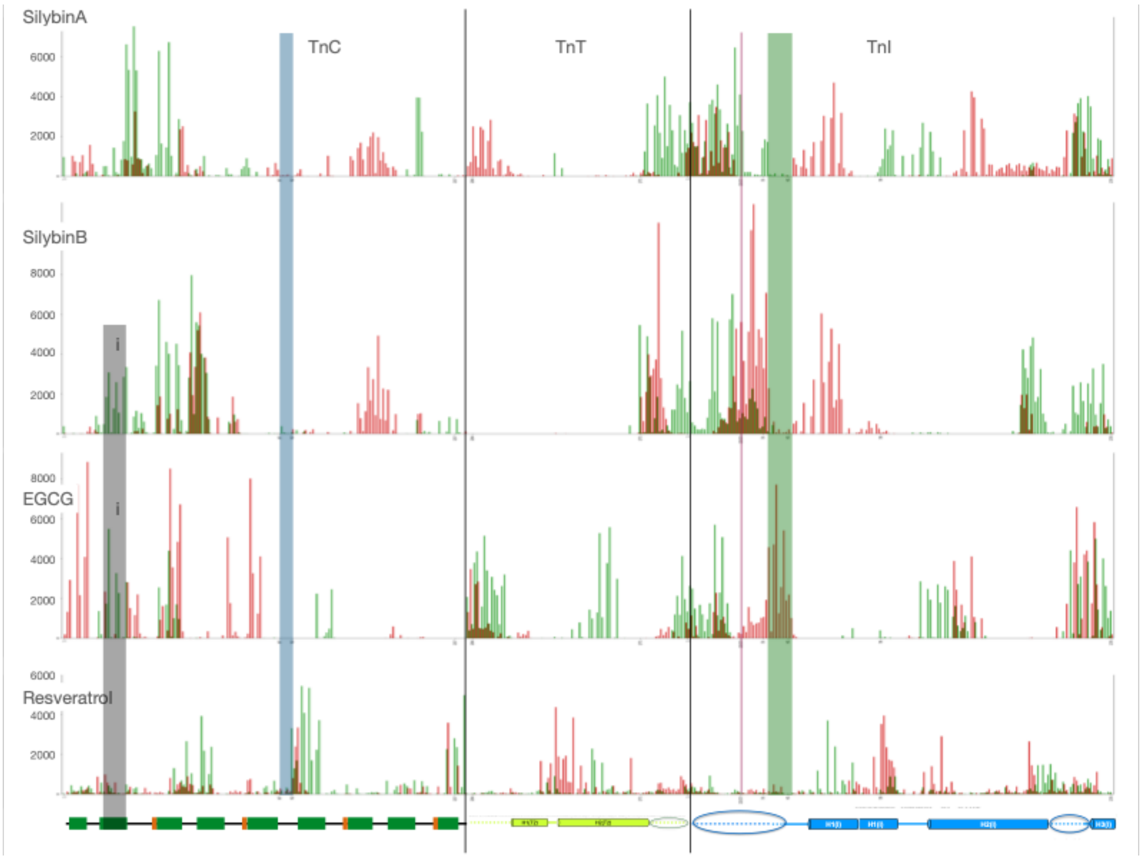

7B

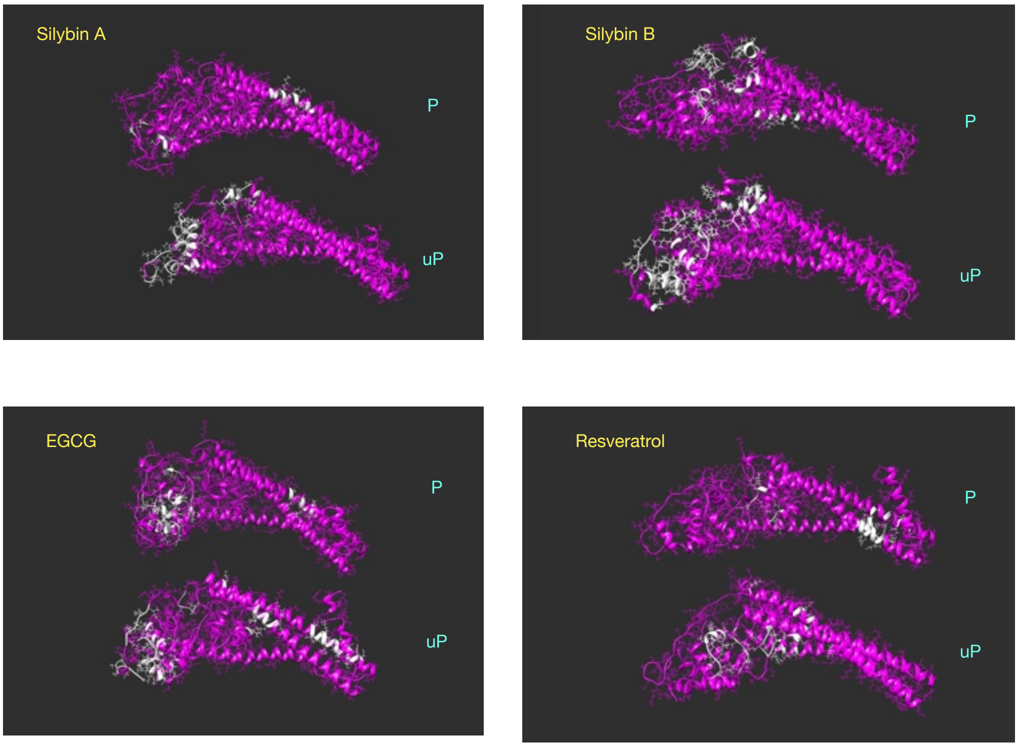

Hotspots: points on troponin where occupancy of ligand exceeds 10% averaged over the 5 x 1500 ns MD runs are shown in white.

7C

For movies follow this link.

https://www.dropbox.com/scl/fo/zq4ttu6kpub3kcethaseu/AHBbV-mwDr6dsSJIr-mEV5A?rlkey=fshl5nivn68j8nrnjq6n2trgh&dl=0

SUPPLEMENT 8

snapshots from single 1500ns MD trajectories, 7500 total frames

Silybin B

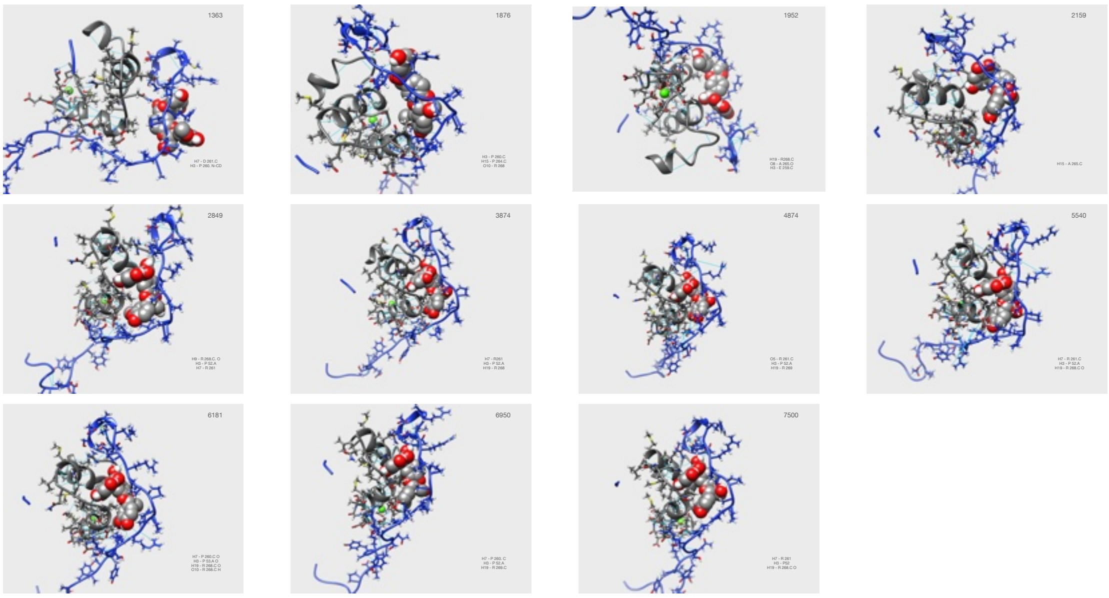

Silybin A

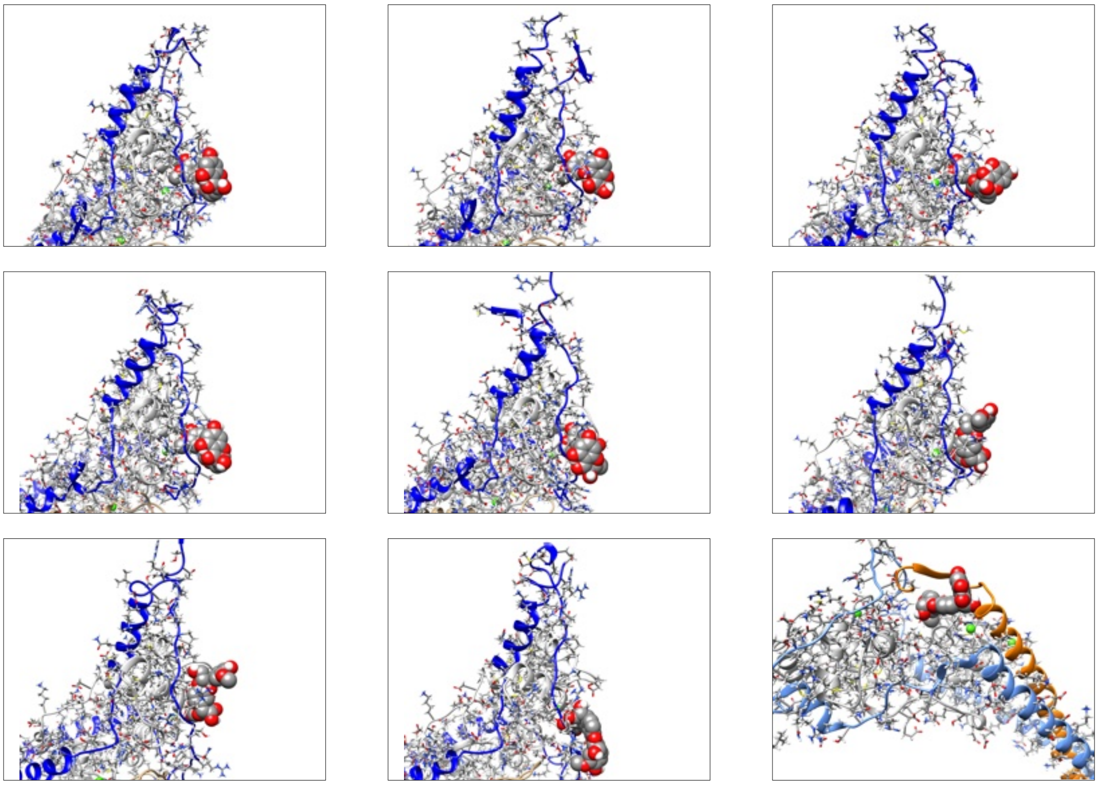

EGCG

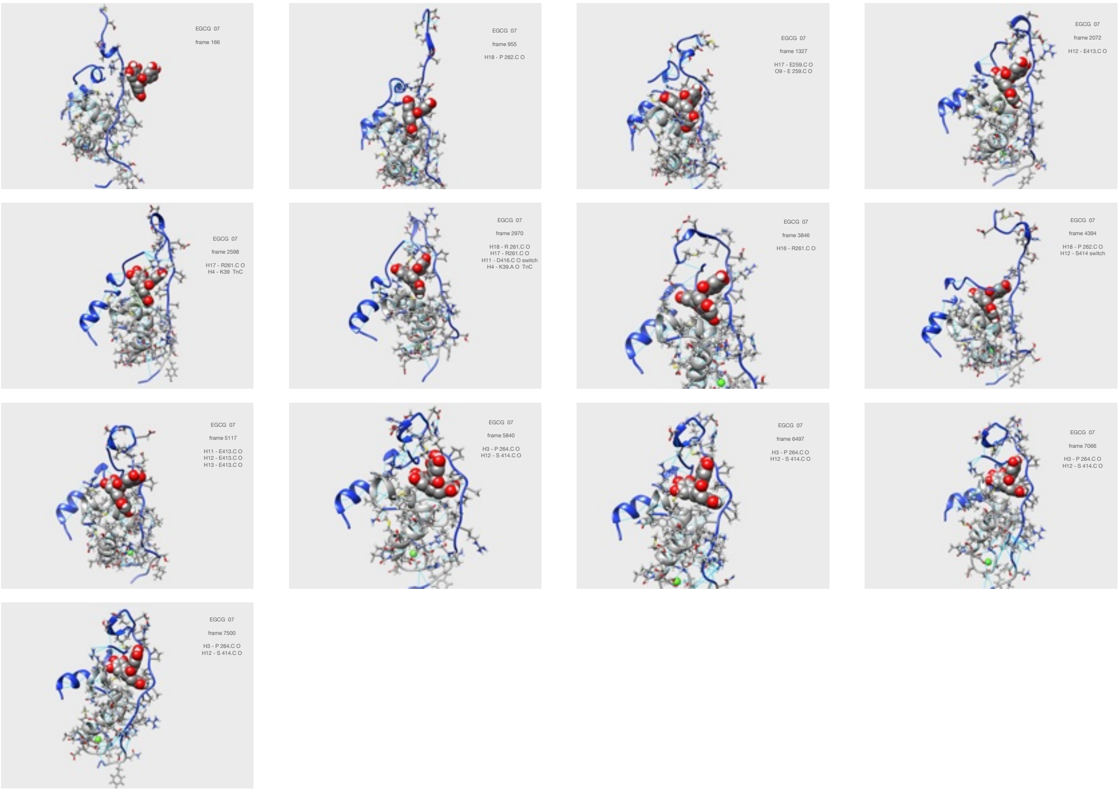

Resveratrol

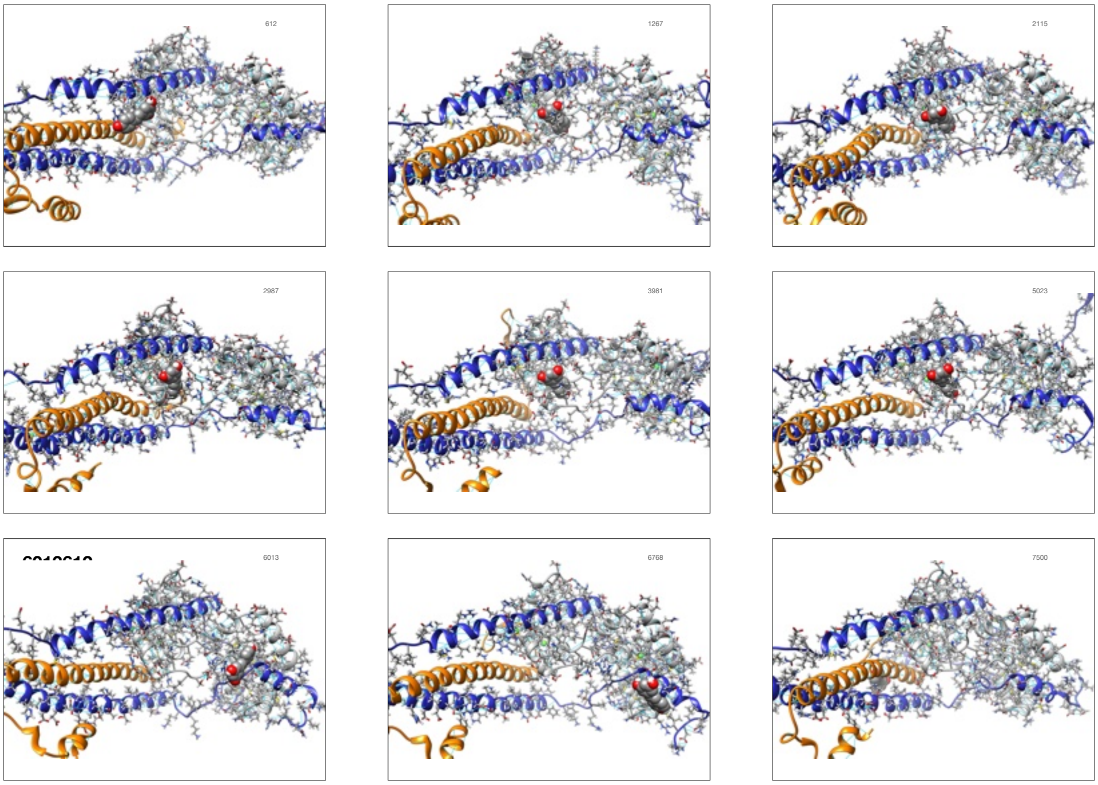

ECG

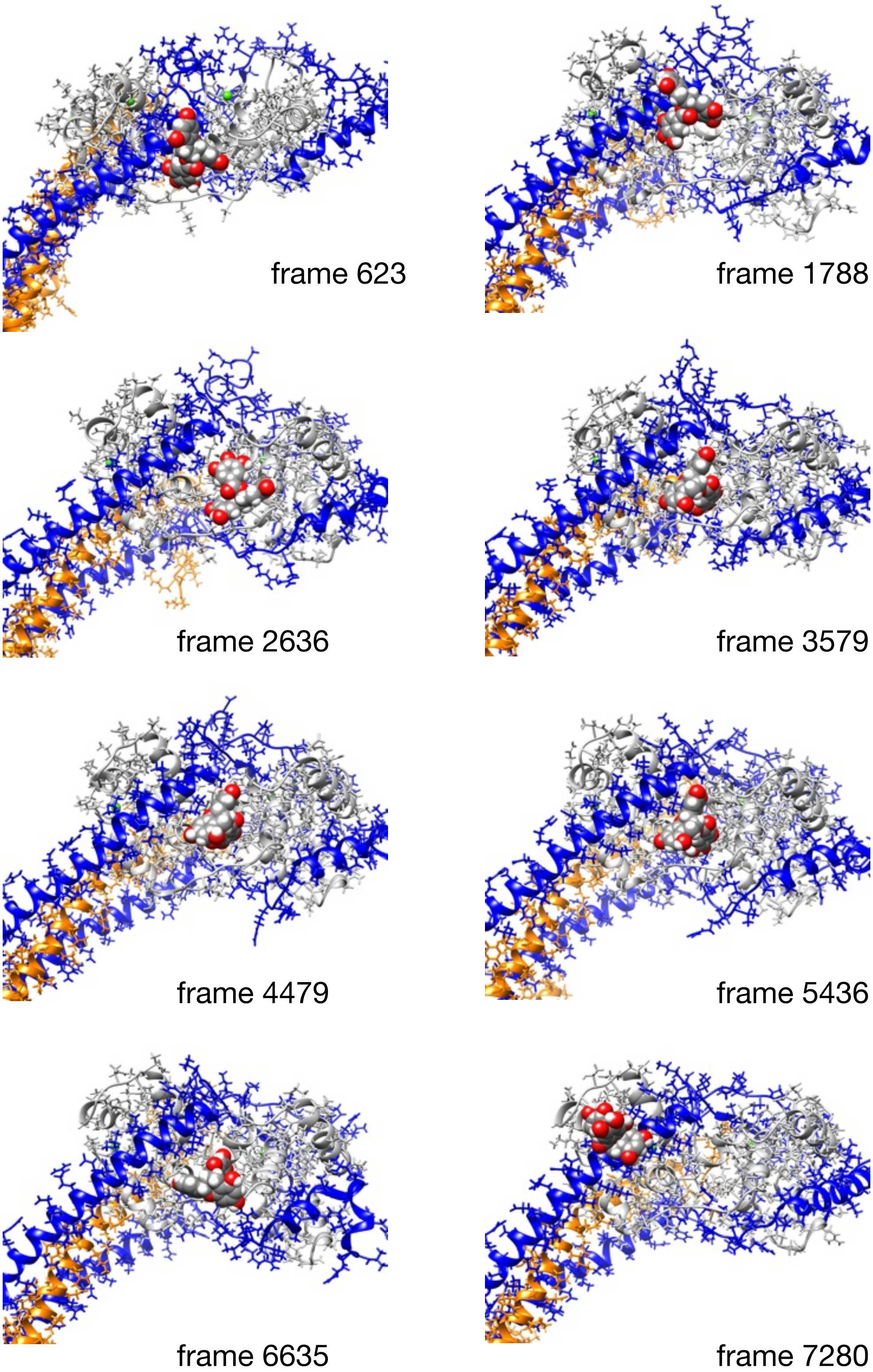

frame 623
frame 1788
frame 2636
frame 3579
frame 4479
frame 5436
frame 6635
frame 7280
